# Prevalence and determinants of lymphedema in newly diagnosed Nigerian breast cancer patients using bioimpedance estimations

**DOI:** 10.3332/ecancer.2023.1506

**Published:** 2023-02-09

**Authors:** Funmilola Wuraola, Olalekan Olasehinde, Matteo Di Bernardo, Patrick Akinyemi, Israel Owoade, Tajudeen Mohammed, Adewale Aderounmu, Samson Ogunleye, Adeoluwa Adeleye, Mary Ogunyemi, Gregory Knapp, Peter Kingham, Olusegun Alatise

**Affiliations:** 1 Department of Surgery, Obafemi Awolowo University, Ile-Ife, Nigeria; 2 Department of Surgery, Obafemi Awolowo University Teaching Hospitals Complex, Ile-Ife, Nigeria; 3 African Research Group for Oncology, Obafemi Awolowo University Teaching Hospitals Complex, Ile-Ife, Nigeria; 4 Department of Surgery, Division of General Surgery, Dalhousie University, Halifax, Nova Scotia, NS B3H 4R2, Canada; 5 Memorial Sloan Kettering Cancer Center, New York, NY 10065, USA

**Keywords:** breast, cancer, lymphedema, bioimpedance

## Abstract

**Background:**

Breast cancer-related lymphedema (BCRL) is common and has significant impact on quality of life. Very little is known about BCRL in sub-Saharan Africa. Generally, BCRL has been mostly evaluated post treatment, with very limited data on the prevalence of pre-treatment BCRL at baseline. This study presents the prevalence and clinical associations of lymphedema among newly diagnosed, treatment-naive breast cancer patients in a Nigerian cohort using bioimpedance estimations.

**Methods:**

Consecutively consenting, newly diagnosed, treatment-naive breast cancer patients were assessed for upper limb lymphedema using bioimpedance measurements of the extracellular fluid and the single-frequency bioelectrical impedance analysis value at 5 kHz. Patients were classified as having lymphedema if there was >10% difference in arm measurements or if the ratios of the arm measurements were >3 SD above a normative mean generated from representative controls. Regression analysis was performed to determine clinical variables associated with lymphedema.

**Results:**

There were 154 breast cancer patients with a median age of 47 (40.0–56.8) years and a body mass index of 27 (23.5–30.9) kg/m^2^. The majority (70%) had stage III disease. All measurements were significantly higher in cases than controls. Using various definitions, the prevalence of lymphedema was between 11.7% and 14.3%. Various clinical variables relating to clinical stage were significantly associated with lymphedema.

**Conclusion:**

The predominance of locally advanced disease in the Nigerian setting is associated with high pre-treatment lymphedema rates. This may set the stage for higher rates in the post-operative setting. Management of lymphedema should be incorporated into the treatment planning.

## Introduction

Breast cancer-related lymphedema (BCRL) is a common complication of breast cancer treatment characterised by an abnormal collection of protein-rich fluid in the interstitial space. Lymphedema can occur following the disruption of the lymphatic drainage from the ipsilateral arm during axillary lymph node staging/resection [1–3]. There is significant heterogeneity in the reported incidence of lymphedema (2%–65%), which is largely attributable to highly variable diagnostic criteria.

Lymphatic disruption may result from extensive nodal metastasis, axillary surgery or irradiation [4–6]. Some patient-related factors such as obesity and recurrent cancer have also been implicated as predisposing factors [1, 2]. More recent data from the United States show some compelling evidence implicating race as a risk factor for developing lymphedema. Black women were observed to have higher odds of developing lymphedema compared to non-African American women [7].

Lymphedema, particularly in severe cases, can be challenging to manage and can have a significant impact on a number of quality of life indicators [5]. Most of the available treatment modalities aim to limit its progression and/or symptomatology as there is yet no proven curative treatment [8–10]. The prevention of lymphedema is considered the most pragmatic approach whenever feasible. To some extent, this has been made possible by the development of effective therapies and early detection of breast cancer, which has enabled the avoidance or de-escalation of axillary surgery.

In Nigeria, as in many parts of sub-Saharan Africa, the subject of lymphedema has been understudied with very limited data on its prevalence and management. The recent data implicating race as a risk factor, coupled with the high prevalence of locally advanced disease with nodal metastasis at the time of initial presentation are compelling reasons to evaluate this subject in the Nigerian setting. Even in early-stage disease, axillary management often entails axillary dissection in Nigeria, given the limited expertise and infrastructure for sentinel lymph node biopsy [11–13].

Anecdotally, lymphedema appears to be a common problem among newly diagnosed breast cancer patients in the Nigerian setting. To quantify this observation, the African Research Group for Oncology (ARGO) started collecting prospective data on lymphedema following modified radical mastectomy in Nigeria. This study presents the prevalence of lymphedema among newly diagnosed breast cancer patients prior to commencement of treatment. It is believed that this work will form an important building block for designing tailor-made interventions targeting BCRL in Nigeria.

## Methods

Consecutive, newly diagnosed breast cancer patients presenting to Obafemi Awolowo University Teaching Hospital over a 3-year period (2019–2021) were recruited for enrolment in this study. The hospital has a prospectively maintained breast cancer database with over a 1,000 patients. The database captures clinical, pathological and treatment-related details with a standardised follow-up protocol that captures treatment outcomes.

### Patients

The patient population in this study included newly diagnosed, treatment-naïve, histologically confirmed breast cancer patients. Relevant clinical details were obtained, including sociodemographic characteristics, anthropometric variables (e.g. weight and height), histopathology and stage at presentation. Patients with bilateral disease at presentation and those with previous breast or axillary operations were excluded.

### Controls

Healthy individuals from the community who responded to an invitation for routine bioimpedance measurements served as controls. These controls were randomly matched in a 1:1 fashion to cases with age being less than or equal to 2 years apart, and body mass index (BMI) being less than or equal to 1 standard deviation (SD) of the BMI of the cases (5.5 kg/m^2^). Given that healthy controls did not present with a malignancy, the arm with the higher bioimpedance measurement was used as the affected side and compared to the measurement with the smaller value employed as the unaffected side.

### Bioimpedance measurements

Patients and controls had bioimpedance assessments using the Inbody 770-2.0® (Biospace, Seoul, South Korea) bioimpedance machine. All measurements were performed by a trained staff using the same machine according to the manufacturer’s guide. Each participant stood barefooted on the two footplates with the heels on the rear sole electrodes and the front parts on the front electrodes. The two arms were kept away from the body with the four-fingers wrapped around the surface of the bottom hand electrode and the thumb on the oval electrode. Details of the bioimpedance measurements were subsequently presented on the screen and exported for analysis.

The bioimpedance estimations of the extracellular fluid (ECF) volume and single-frequency bioelectrical impedance analysis value at 5 kHz for both upper extremities were used in the determination of lymphedema estimates in this study.

The first set of lymphedema-related measurements was derived from the extracellular water (ECW) measurements for each arm. Comparing the affected to the unaffected side, the ratio of ECW of affected versus unaffected arm (continuous), and difference in ECW of >10% between the affected and unaffected arm (binary) were calculated for both cases and controls.

The second set of lymphedema-related measurements was derived from 5 kHz impedance measurements for each arm. Comparing the unaffected side to the affected side, we derived the ratio of 5 kHz impedance measurement of unaffected versus affected arm (continuous), and difference in 5 kHz measurements > 10% (binary) between unaffected versus affected arm.

### Definition of lymphedema

Lymphedema was defined using various diagnostic criteria available in the literature [3, 14–18]:

- A difference of >10% in the ECW measurements of affected versus unaffected arm.
- A difference of >10% in the 5 kHz impedance measurements of unaffected versus affected arm.
- ECW ratio (affected versus unaffected arm) more than 3 SDs above the normative data (derived from controls).
- 5 kHz impedance ratio (unaffected versus affected arm) more than 3 SDs above the normative data (derived from controls).

### Statistical analysis

All statistical analysis was done in R-studio 1.3.1073, using R v 4.0.2.

One-sided *t*-tests were used to compare measurements (continuous or binary) between cases and controls. Based on previously published data, a number of quantitative indicators were employed to understand the prevalence of lymphedema at presentation (>10% difference in measurements and bioimpedance ratios > 3 SD above controls), and if such measurements are associated with clinical data. We examined the relationship between a number of clinical variables and the presence of lymphedema, including affected side (right, left), sex (female, male), age (years), BMI (kg/m^2^), body surface area (BSA) (m^2^), waist circumference (cm), hip circumference (cm), mass size (cm), clinical T stage (1, 2, 3, 4), clinical N stage (1, 2, 3), clinical M stage (0, 1) and overall clinical stage (I, II, III, IV). All regression models were performed as univariate due to the close inter-relatedness of these clinical variables.

For cases only, linear regression models were used for continuous measurements to understand how various clinical variables may be statistically relevant in predicting lymphedema. This was done for all continuous lymphedema measurements that were statistically significant (in comparing cases and controls) in the methodologies above. Logistic regression models were used for binary measurements in a similar fashion. This was done for all binary lymphedema measurements that were statistically significant (in comparing cases and controls) in the tests above. The lymphedema measurement that showed the lowest incidence (difference in ECW > 10%) was selected for the univariate analysis. This was quite representative of the clinical associations that were observed with the other lymphedema measurements. For all statistical analysis, *p* value was set at 0.05.

## Results

### Baseline characteristics

A total of 154 Nigerian breast cancer patients were included in this study (Table 1). From a total of 783 healthy volunteers, 148 healthy controls were matched to the cases. The median age and BMI of the breast cancer patients were 47 (40.0–56.8) years and 27 (23.45–30.97) kg/m^2^, respectively. In the control group, median age was 46 (40–53) years while BMI was 27.75 (23.62–30.55) kg/m^2^. The median tumour size was 8.5 (6–14) cm with the majority (70%) having stage III disease.

### Bioimpedance estimated prevalence of lymphedema

The proportion of patients with lymphedema based on the difference of >10% in the ECW arm measurements was 11.7% while 12.3% of the patients were adjudged to have lymphedema using ECW ratio of more than 3 SDs above the normative data (>1.091).

Using a single-frequency bioimpedance measurement at 5 kHz, the proportion of patients with lymphedema was 13.6% based on a difference of >10% between the arms, while it was 14.3% using the 5 kHz impedance ratio greater than 3 SDs above the normative data (>1.095) (Table 2).

### Comparison of cases and controls

The prevalence of lymphedema among breast cancer patients was significantly higher than in controls (11.7%–14.3% versus 0%–2.0%). A comparison of all but one bioimpedance measurements (difference in 5 kHz impedance) between cases and controls showed statistically significant differences in all measured quantities (Table 3).

### Clinical determinants of lymphedema

Of the clinical variables tested, mass size, nodal staging and overall clinical staging were the only factors that were significantly associated with the development of lymphedema. Age, BMI and other clinical variables were not significantly associated with the development of lymphedema (Table 4).

## Discussion

BCRL has been mostly evaluated following treatment. This study focused on disease-related lymphedema using bioimpedance measurements among newly diagnosed, treatment-naive breast cancer patients. Evaluating the prevalence of lymphedema at baseline provides some insight into preoperative factors that might be associated with its occurrence and also allows for comparison of pre-and post-treatment values to determine whether treatments worsen or resolve it. For the first time in sub-Saharan Africa, we report a baseline incidence of lymphedema of 11.7%–14.3% in breast cancer patients at presentation, particularly in patients presenting with locally advanced disease, which comprised the majority of the study population. The high prevalence of lymphedema justifies routine surveillance at the time of initial presentation as early diagnosis may prompt the inclusion of preventive and therapeutic measures in the treatment plan. Currently, there are no clear-cut guidelines or recommendations on the management of lymphedema in the pre-operative setting and how it might affect decision-making regarding surgery and irradiation. Presumably in these patients, the surgical plan will be individualised to minimise further lymphatic disruption and worsening of lymphedema post-operatively.

Traditionally, arm circumference measurement has been the most commonly used method of diagnosing lymphedema. The most common criterion for lymphedema diagnosis using arm circumference is greater than or equal to 2 cm inter-limb difference at any single location, or greater than or equal to 200 mL volume difference. Given that arm circumference measurements assess the volume of the entire limb, it might be affected by changes in muscle and fat mass [15, 19]. This study adopted the use of bioimpedance measurements given its proven validity and improved accuracy over routine arm measurement. Bioimpedance analysis measures body response to applied electrical current and calculates the body fluid volume [14, 20]. The technology can differentiate ECF from total limb volume. Bioimpedance is known to diagnose lymphedema at least 10 months before it becomes clinically apparent [21]. It also compares favourably with optoelectric perometry which uses infrared light emitted from a frame that passes over the limb [17, 22, 23]. Although considered the gold standard, the cost of perometry limits its applicability in resource-constrained settings. Bioimpedance, which is less expensive, is therefore a more attractive option especially in resource-limited settings. In sub-Saharan Africa, the use of bioimpedance for diagnosing lymphedema is quite novel and to date there is no data for comparison. Various diagnostic criteria exist in the literature for defining lymphedema. With the use of bioimpedance, the commonly used criteria are the ECW and single-frequency bioimpedance measurements used in this study. These parameters have been evaluated in various studies with good diagnostic accuracy [3, 16]. The lymphedema rates obtained from the two parameters either by comparing the affected to the unaffected arm or by comparing ratios of patients to controls yielded comparable results.

A baseline prevalence of lymphedema > 10% prior intervention is quite concerning. The patient population in this study may partially explain this high rate. The majority had locally advanced lesions with axillary involvement at presentation (70% had stage III disease). Although the data on pre-operative lymphedema is limited, it appears to be much less common in contemporary series from North America. In a large prospective cohort of women undergoing pre- and post-operative lymphedema screening at Massachusetts General Hospital, only 2.5% of women had an ipsilateral arm volume measurement that was >10% compared to the contralateral side [24]. Early diagnosis of breast cancer reduces the risk of axillary involvement and consequently the risk of lymphedema at the time of presentation. This study clearly identifies three variables: mass size, N stage and overall stage as positive predictors of lymphedema. All of these indicate that advanced disease stage is correlated to and may increase the risks of lymphedema at presentation. Different from several other studies on BCRL, this analysis found no association between lymphedema and BMI [25]. Such associations have been demonstrated among patients evaluated post-treatment rather than newly diagnosed patients as presented in this series.

The need to explore the subject of lymphedema among blacks is substantiated by recent data from the United States. In a cohort of 276 patients who had undergone axillary lymph node dissection, a lymphedema rate of 24.7% was reported, with black women having a 3.8-fold increased risk of developing lymphedema compared to white women after controlling for known-confounders [7]. Our study represents the first step in exploring the subject in details among a homogenously black population. The post-operative lymph oedema rate in our cohort is being evaluated in an ongoing study, and this will be compared with earlier studies. A study comparing lymphedema rates in stage and treatment comparable patients across different races and regions of the world using the same surveillance technique might be an interesting undertaking.

Findings from this study were based solely on bioimpedance measurements. Hence, we did not determine the proportion of patients with clinically significant lymphedema presenting with arm symptoms such as pain, heaviness or swelling. This has been taken into consideration in an ongoing longitudinal study evaluating the prevalence and determinants of BCRL in the same institution. It will also be interesting to know how pre-treatment lymphedema relates to the development of post-treatment lymphedema.

## Conclusion

This study has provided evidence that suggests a high prevalence of pre-treatment BCRL in a Nigerian cohort. The findings of this study provide a strong basis to explore this subject in greater detail.

## Funding

This study did not receive any funding for the execution of this project.

## Conflicts of interest

The authors have no relevant financial or non-financial conflicts of interest.

## Author contributions

All authors contributed to the study conception, design and execution.

## Data availability

The datasets used during the current study are not publicly available. It can however be made available from the corresponding author on reasonable request.

## Ethics approval and consent to participate

This study received necessary approval from the ethical review board. All procedures were carried out in accordance with relevant guidelines and regulations. Consent was obtained from participants before participation.

## Figures and Tables

**Table 1.** Clinical characteristics of cases and controls.

Breast cancer patients (*n* = 154)		
	*n*/median (IQR)	Proportion
Side		
Right	75	49%
Left	79	51%
Sex		
Female	152	99%
Male	2	1%
Age	47 (40.0–56.8)	
BMI	27 (23.5–30.9)	
BSA	1.7 (1.6–1.9)	
Waist circumference (cm)	37 (33.5–39.0)	
Hip circumference (cm)	40 (38–44)	
Mass size (cm)	8.5 (6–14)	
Clinical T stage		
1	2	1%
2	19	12%
3	32	21%
4	100	65%
Clinical N stage		
1	26	17%
2	99	64%
3	28	18%
Clinical M stage		
0	146	95%
1	7	5%
Clinical stage		
I	2	1%
II	36	23%
III	108	70%
IV	7	5%
Controls (*n* = 148)		
	*n*/median (IQR)	Proportion
Age	46 (40–53)	
BMI	27.75 (23.6–30.6)	

IQR: interquartile range

**Table 2.** Prevalence of lymphedema.

Estimated bioimpedance measurement	Difference in ECW > 10%	Ratio of ECW > 3 SD of normative value	Difference in 5 kHz impedance > 10%	Ratio of 5 kHz impedance > 3 SD of normative value
Prevalence	11.7%	12.3%	13.6%	14.3%

**Table 3.** Comparison of cases and controls.

ECW measurements	Cases	Controls	*p*-value
Difference in ECW (L)	0.0342 (0.02–0.04)	0.0158 (0.01–0.02)	0.042^a^
Ratio of ECW	1.059 (0.098–1.05)	1.023	0.025^a^
Difference in ECW > 10%	18 (11.7%)	2 (3.5%)	0.0003^a^
5 kHz impedance measurements			*p-*value
Difference in 5 kHz impedance	15.07 (6.3–19.75)	8.896 (3.27–11.38)	0.051
Ratio of 5 kHz impedance	1.063 (0.98–1.05)	1.025 (1.01–1.03)	0.022^a^
Difference in 5 kHz impedance > 10%	21 (13.64%)	3 (2.01%)	0.0002^a^

a Statistically significant (*p* < 0.05)

**Table 4.** Clinical determinants of lymphedema.

Clinical variable	Difference in ECW > 10% Estimated coefficient (p-value)
Side (Right = 0, Left = 1)	0.4520 (0.378)
Sex (Female = 0, Male = 1)	-14.559 (0.993)
Age (years)	−0.004208 (0.8357)
BMI (kg/m^2^)	−0.05238 (0.303)
BSA (m^2^)	−2.415 (0.0941)
Waist circumference (cm)	−0.07539 (0.190)
Hip circumference (cm)	−0.11615 (0.0575)
Mass size (cm)	0.10281 (0.00454)^a^
Clinical T stage	−0.11615 (0.0575)
Clinical N stage	1.2807 (0.00534)^a^
Clinical M stage	1.1787 (0.179259)
Overall clinical stage	1.6385 (0.007006)a

a Statistically significant (*p* < 0.05)
